# Fatal Pulmonary Embolism following Achilles Tendon Repair: A Case Report and a Review of the Literature

**DOI:** 10.1155/2013/401968

**Published:** 2013-05-30

**Authors:** Asim M. Makhdom, Simon Garceau, Ronald Dimentberg

**Affiliations:** ^1^Division of Orthopaedic Surgery, McGill University, 1529 Cedar Avenue, Montreal, QC, Canada H3G 1A6; ^2^Department of Orthopaedic Surgery, King Abdulaziz University, Jeddah 21589, Saudi Arabia; ^3^Division of Orthopaedic Surgery, University of British Columbia, Vancouver, BC, Canada V5Z 1M9

## Abstract

Deep venous thrombosis (DVT) is a significant source of morbidity in orthopaedic surgery. It can progress to a pulmonary embolism, a significant source of mortality. Up to date, patients with Achilles tendon rupture routinely do not receive DVT chemical prophylaxis. We are presenting a case of fatal pulmonary embolism after a surgically treated Achilles tendon rupture in a forty-two-year-old male healthy patient. In the current body of the literature, the reported incidence of DVT after Achilles tendon rupture is highly variable ranging from less than 1% to 34%, and there is a disagreement in the international guidelines regarding the need of chemical DVT prophylaxis with this type of injury. Further research needs to be conducted to investigate the risks and benefits of chemical DVT prophylaxis following Achilles tendon rupture. For low-risk patients, the use of milder forms of prophylaxis such as aspirin should also be explored.

## 1. Introduction

Deep venous thrombosis (DVT) is an important source of morbidity in orthopaedic surgery. It can progress to pulmonary embolism (PE), a significant source of mortality [1, 2]. Both operative and nonoperative treatments of Achilles tendon rupture include a period of immobilization which is a well-documented risk factor for DVT [2]. However, the most recent antithrombotic guidelines (published by the American College of Chest Physicians) suggested no DVT prophylaxis for this type of injury [3]. In this report, our primary aim is to present a case of fatal pulmonary embolism following surgical Achilles tendon repair. The secondary aim is to review the literature with regard to the incidence of DVT and PE after Achilles tendon rupture.

## 2. Case Report

A forty-two-year-old male highly active patient presented to the orthopaedic clinic with a five-week history of a painful left heel that has been brought on suddenly during folk dancing. The patient was able to walk with a limp but had notable functional impairment and was unable to climb a flight of stairs. He continued working as a microbiologist and refrained from seeking any medical attention until his wife eventually urged him to consult an orthopaedic surgeon. He presented with no known medical conditions and was not on any medication at that time. Two years prior to his injury, he had used isotretinoin for acne. This medication was used for a period of two months. He was not a smoker and had a body mass index of 25.8 Kg/m^2^. Surgical history was unremarkable with the exception of an arthroscopic partial meniscectomy for a right knee meniscal tear four years prior to presentation. The cardiovascular family history included the patient's grandfather who died of myocardial infarction at the age of sixty-five. No hereditary prothrombotic conditions were known to afflict the patient or his relatives. On physical examination, the patient was found to have a 3 cm palpable gap of the left Achilles tendon associated with a positive Thompson test. The patient agreed to undergo surgical repair and was placed on the operative list for the following day. He was induced under general anaesthesia, and a tourniquet was inflated for thirty-nine minutes. The tendon was sutured using a modified Kessler technique. The patient was subsequently immobilized in a three-sided plaster cast in a plantar flexed position and was sent home the following day on crutches.

Approximately ten days postoperatively, the patient was at home when he developed a cough associated with symptoms of dyspnea. This lasted for thirty minutes then subsided. The patient did not go to work the following day claiming he felt unwell. Two days after this event, he felt better and presented to his follow-up appointment at the orthopaedic clinic. While he was in the examination room, he collapsed and became unresponsive. Cardiopulmonary resuscitation was started immediately by the treating orthopaedic surgeon and his assistant. This was continued until arrival of the paramedic services. He was then transported to the nearby emergency department where an EKG revealed pulseless electrical activity. Cardiopulmonary resuscitation was continued for a total of forty-five minutes but proved unsuccessful. An autopsy report later revealed massive bilateral acute pulmonary (saddle) embolus.

## 3. A Literature Review and Discussion

The incidence of deep venous thrombosis after Achilles tendon rupture was highly variable in the literature. The reported incidence ranges from less than one percent to thirty-four percent (Table 1) [4–12]. Lapidus et al. noted a DVT incidence of 34% in ninety-one patients after surgical repair with no reported PEs [6]. Nilsson-Helander et al. studied ninety-five patients and recorded similar DVT incidences after Achilles tendon rupture in both the operative and nonoperative study groups (34%). They also found that 3% of these patients developed PEs [7]. Furthermore, Healy et al. noted a DVT incidence of 6.3% and a PE incidence of 1.5% in 208 patients [8]. They found this rate similar to that of PE in patients having undergone elective hip surgeries. They suggest that this may be secondary to a lack of DVT prophylaxis in patients with lower limb immobilization after Achilles tendon rupture. Saragas and Ferrao found DVT incidence of 5.7% in 88 patients and one patient had developed near fatal pulmonary embolism [12]. Recently, Makhdom et al. found that the symptomatic DVT incidence after Achilles tendon rupture repair was 23.47% in 115 patients [10]. The authors noted that one-third of the 23.47% had developed DVT prior to their surgery. On the other hand, Patel et al. recently performed a retrospective analysis, which looked at 1172 patients for the incidence of DVT and PE with Achilles tendon rupture. They found an incidence of 0.43% and 0.34%, respectively [9]. Hence, there is a discrepancy in the reported incidence of DVT in patients with Achilles tendon rupture in both the operative and nonoperative groups. The reasons for this difference remain unclear. It seems reasonable to conclude that this variation, at least partly, stems from the different study designs; some studies reported symptomatic DVTs, whereas others reported both symptomatic and asymptomatic DVTs.

In the literature, there are few case reports documenting Achilles tendon rupture with subsequent DVT and PE. One such report documented a PE in a patient with a prothrombotic condition [13], and two were in individuals believed to be generally healthy [14, 15]. Furthermore, the PE was fatal in one patient who had undergone conservative management for Achilles tendon rupture [14]. To our knowledge, this is the first case of fatal PE following surgical repair of an Achilles tendon rupture in the English medical literature.

A number of factors are believed to contribute to the risk of developing DVT/PE with cast immobilization of the lower limb following injury. These include trauma, prolonged immobilization, and surgery [11]. However, several authors disagree that surgery for Achilles tendon rupture is a risk factor for DVT/PE [7, 9]. Although our patient had some of these risk factors, we recognize the possibility that he may have developed an asymptomatic DVT prior to his surgery. Additionally, in our report, we noted that our patient had a history of isotretinoin use. Although there are several case reports suggesting the possibility of an increased risk of thromboembolic events with the use of this medication [16], most of these case reports were in patients suffering from leukemia treated with all-trans-retinoic acid [17–19]. No studies to date have demonstrated such an association [20].

Thromboembolism has been reported with many orthopaedic procedures [21, 22]. Although in the hip and knee there are clear consensus guidelines regarding DVT prophylaxis [3], the optimal DVT/PE prophylaxis following below the knee injury requiring immobilization remains unclear. There is recent evidence from a Cochrane meta-analysis which has noted a decreased incidence of symptomatic DVT from 2.5% to 0.3% between patients randomized to placebo versus LMWH [21] in this population. On the other hand, others believe the use of routine prophylaxis to be unwarranted due to a low frequency of symptomatic DVTs and PEs [9]. International consensus guidelines vary significantly on recommendations for injuries below the knee requiring immobilization [3, 22]. Further research should therefore be conducted to investigate the risks and benefits of chemical DVT prophylaxis in patients following Achilles tendon rupture. For low-risk patients, the use of milder forms of prophylaxis such as aspirin should also be explored [8].

## Figures and Tables

**Table 1:** Reported incidence of deep vein thrombosis and pulmonary embolism in patients treated surgically (S) and nonsurgically (N-S) for Achilles tendon rupture.

Author(s) and date of publication	Number of patients included in the study	Surgical intervention (S)/nonsurgical intervention (N-S)	Incidence of deep vein thrombosis (%)	Incidence of pulmonary embolism (%)
Lo et al. (1997) [4]	949	S = 701/N-S = 248	S = 0.14/N-S = 1.6	S = 0/N-S = 0.40
Ingvar et al. (2005) [5]	196	N-S = 196	N-S = 3.6	N-S = 0.51
Lapidus et al. (2007) [6]	91	S = 91	S = 35	S = 0
Nilsson-Helander et al. (2009) [7]	95	S = 49/N-S = 46	S = 34	S = 3.2
Healy et al. (2010) [8]	208	S = 208	S = 6.3	S = 2.4
Saragas and Ferrao (2011) [12]	88	S = 88	S = 5.7	S = 1.1
Patel et al. (2012) [9]	1172	S = 472/N-S = 700	S = 0.42/N-S = 0.43	S = 0.21/N-S = 0.43
Makhdom et al. (2013) [10]	115	S = 115	S = 23.47	S = 0.8
